# Five new species of the genus *Ischnothyreus* Simon, 1893 from Singapore

**DOI:** 10.3897/zookeys.618.9451

**Published:** 2016-09-19

**Authors:** Yanfeng Tong, Joseph K. H. Koh, Xiujiao Tong, Shuqiang Li

**Affiliations:** 1 Life Science College, Shenyang Normal University, Shenyang 110034, China; 2 Southeast Asia Biodiversity Research Institute, Chinese Academy of Sciences, Menglun, Mengla, Yunnan 666303, China; 3 National Biodiversity Centre, National Parks Board, 259598, Singapore; 4 Institute of Zoology, Chinese Academy of Sciences, Beijing 100101, China

**Keywords:** Diagnosis, goblin spider, morphology, taxonomy, type

## Abstract

Five new species of the genus *Ischnothyreus* are reported from Singapore: *Ischnothyreus an* Tong & Li, **sp. n.**, *Ischnothyreus brunneus* Tong & Li, **sp. n.**, *Ischnothyreus dactylinus* Tong & Li, **sp. n.**, *Ischnothyreus poculum* Tong & Li, **sp. n.** and *Ischnothyreus tectorius* Tong & Li, **sp. n.** Morphological descriptions and illustrations are given for all new species.

## Introduction

With a population of 5.5 million people packed in a total land area measuring only 719 km^2^, the Republic of Singapore is one of the most urbanized countries in the world. Yet, Singapore projects itself as a “city in a garden”, with 9,704 hectares or 13.5% of Singapore still covered with greenery, including 3,375 hectares (4.7%) fully protected as Nature Reserves (National Parks Board 2015). It is thus not surprising that Singapore is still home to a surprising diversity of flora and fauna, with many new species discovered even in recent years.

Out of the 1,628 described species in 113 oonopid genera worldwide, only 129 valid species, currently assigned under 12 genera, have been described from southeast Asia (Li and Lin 2016; World Spider Catalog 2016). The foundation was laid by pioneer arachnologists such as Koch (1873), Simon (1893, 1905, 1907, 1909), and Thorell (1887, 1890, 1895, 1897). After a hiatus of almost a century, the knowledge has been augmented in recent studies including those by Baehr et al. (2012), Eichenberger et al. (2012), Eichenberger and Kranz-Baltensperger (2011), Kranz-Baltensperger (2011, 2012), Thoma et al. (2014), and Tong and Li (2013a, b, c). Among the total of 12 species of Singapore that has been documented in published records, four species were described with Singapore as their type locality. Two of them were described by Simon, viz., *Gamasomorpha camelina* Simon, 1893, and *Xyphinys hystrix* Simon, 1893; and another two by Thoma, viz., *Aposphragisma salweskii* Thoma, 2014 and *Aposphragisma stannum* Thoma, 2014.

The genus *Ischnothyreus* Simon, 1893 can be recognized by the presence of leg spines, the usually small abdominal scutum, the strongly sclerotized male palps, the heavily sclerotized male endites and the winding genital tube in the females (Kranz-Baltensperger 2011). There are currently 84 valid specific names assigned to *Ischnothyreus*, but the presently recognized species may represent only a small fraction of the actual biodiversity (Edward and Harvey 2014). Among these 84 species are 28 recorded from Southeast Asia (World Spider Catalog 2016). Only two of these were recorded in Singapore itself, viz., *Ischnothyreus flagellichelis* Xu, 1989, previously described in China; and a pantropical species *Ischnothyreus peltifer* (Simon, 1891) whose type locality is St. Vincent (Murphy and Murphy 2000; Song et al. 2002).

As no oonopids have been deposited at the Lee Kong Chian Natural History Museum in Singapore, a concerted survey of the oonopid spiders was carried out in Singapore in August 2015, with the support and encouragement of the Singapore National Parks Board. From the many specimens of *Ischnothyreus* collected, neither of the two species of previously recorded from Singapore was recognized. However, we have been able to add five new species of *Ischnothyreus* to the Singapore Oonopidae inventory.

## Material and methods

All the specimens were collected by sifting leaf litter. The specimens were examined using a Leica M205C stereomicroscope. Details were studied under an Olympus BX51 compound microscope. All illustrations were made using a drawing tube and inked on ink jet plotter paper. Photos were made with a Canon EOS 550D zoom digital camera (18 mega pixels) mounted on an Olympus BX51 compound microscope. Vulvae were cleared in lactic acid. Male palps and chelicerae were mounted in Kaiser’s glycerol gelatin. All measurements were taken using an Olympus BX51 compound microscope and are in millimeters.

The following abbreviations are used in the text: ALE = anterior lateral eyes; PLE = posterior lateral eyes; PME = posterior median eyes.

All types of the new species are deposited in Lee Kong Chian Natural History Museum, National University of Singapore (LKCNHM). Other material studied is deposited in Shenyang Normal University (SYNU) in Shenyang, China.

## Taxonomy

### Ischnothyreus an

Taxon classificationAnimaliaAraneaeOonopidae

Tong & Li sp. n.

http://zoobank.org/ACEC0E27-0783-4364-9D15-7EACAEBC161F

Figs 1 , 2 , 3

#### Type material.

**Holotype**: male (LKCNHM), Singapore: Central Catchment Nature Reserve, Alt. 60 m, 1°21'21.7"N, 103°48'3.8"E﻿﻿, August 26, 2015, S. Li and Y. Tong leg. **Paratypes**: 1 male, 5 females (LKCNHM), same data as holotype.

#### Other material studied.

4 females (SYNU-60), Singapore: Central Catchment Nature Reserve, near Mandai Agrotechnology Park, Alt. 46 m, 1°24'53.7"N, 103°47'56.2"E, Sep 1, 2015, S. Li and Y. Tong leg.; 8 females (SYNU-61), Singapore: Central Catchment Nature Reserve, Alt. 46 m, 1°21'13.3"N, 103°48'29.4"E, August 27, 2015, S. Li and Y. Tong leg.; 6 males, 6 females (SYNU-62), Singapore: Central Catchment Nature Reserve, treetop walk, 1°21'13.3"N, 103°48'29.4"E, August 28, 2015, S. Li and Y. Tong leg.; 1 male, 1 female (SYNU-63), Singapore: Central Catchment Nature Reserve, Alt. 39 m, 1°21'17.9"N, 103°47'50.7"E, August 25, 2015, S. Li and Y. Tong leg.; 2 males, 1 female (SYNU-64), Singapore: Central Catchment Nature Reserve, Alt. 39 m, 1°21'17.9"N, 103°47'50.7"E, August 25, 2015, S. Li and Y. Tong leg.

#### Etymology.

The species's name is derived from the Chinese Pinyin "an", meaning dark, which refers to the color of the palp; term in apposition.

#### Diagnosis.

**Figure 1.**
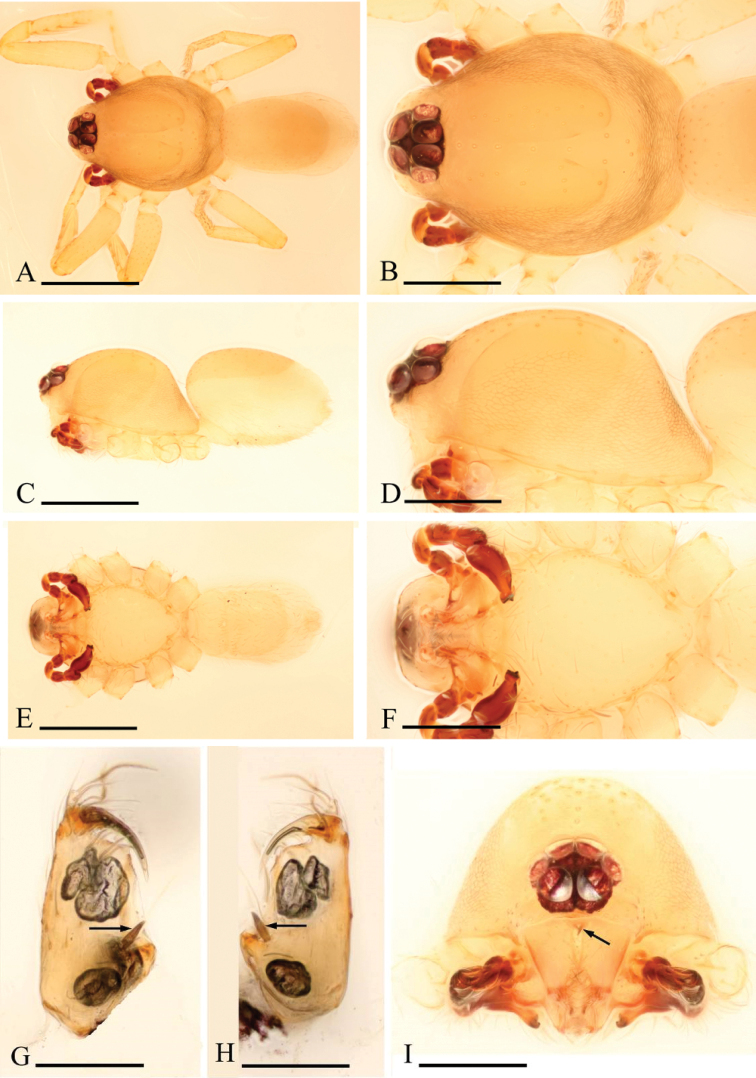
*Ischnothyreus an* sp. n., male. **A, C, E** habitus, dorsal, lateral and ventral views **B, D, F, I** prosoma, dorsal, lateral, ventral and anterior views **G, H** left chelicera, anterior and posterior views. Arrows show the thorn-like protrusion. Scale bars: **A, C, E** = 0.4 mm; **B, D, F, I** = 0.2 mm; **G, H** = 0.1 mm.

**Figure 2.**
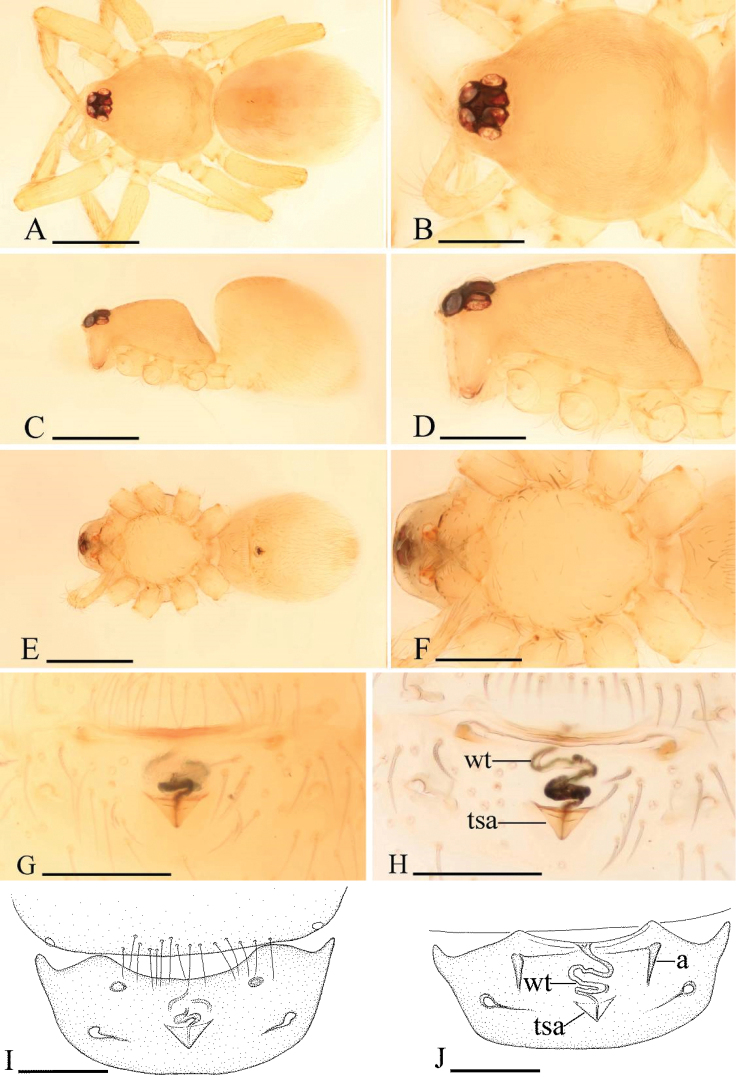
*Ischnothyreus an* sp. n., female. **A, C, E** habitus, dorsal, lateral and ventral views **B, D, F** prosoma, dorsal, lateral and ventral views **G, I** epigastric region, ventral view **H** epigastric region, ventral view (cleared in lactic acid) **J** epigastric region, dorsal view. Abbreviations: a = apodeme; tsa = triangular shaped atrium; wt = winding tube. Scale bars: **A, C, E** = 0.4 mm; **B, D, F** = 0.2 mm; **G–J** = 0.1 mm.

**Figure 3.**
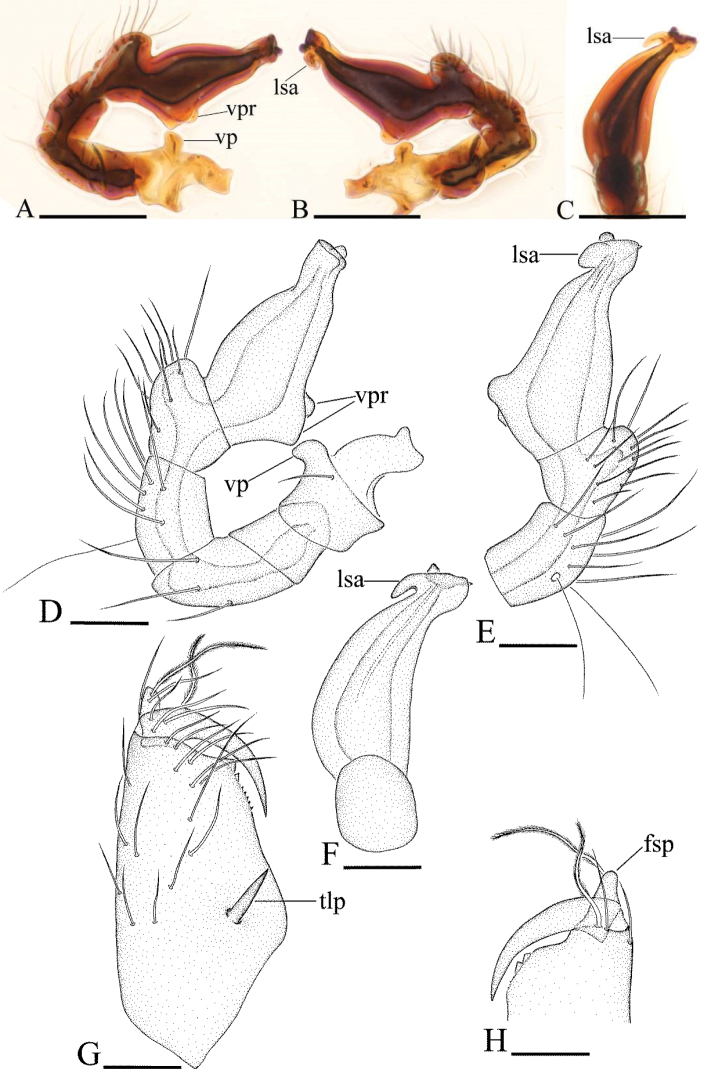
*Ischnothyreus an* sp. n., male. **A, D** left palp, prolateral view **B, E** left palp, retrolateral view **C, F** left palp, dorsal view **G, H** left chelicera, anterior and posterior views. Abbreviations: fsp = finger-shaped sclerotized process; lsa = leaf-shaped apophysis; tlp = thorn-like protrusion; vp = ventral projection; vpr = ventral protuberance. Scale bars: **A–C** = 0.1 mm; **D–H** = 0.05 mm.

The new species is similar to *Ischnothyreus tekek* Kranz-Baltensperger, 2012 in having similar thorn-like protrusion (tlp) on the proximal part of the paturon (Figs 1G, H, 3G and Kranz-Baltensperger 2012: fig. 3D) in male, but can be distinguished from it by the finger-shaped sclerotized process (fsp) at base of fangs (Fig. 3H) in male, and the triangular shaped atrium (tsa) in the female epigastric region (Fig. 2G–J)

#### Description.

Male (holotype). Total length 1.18; carapace 0.65 length, 0.48 width; abdomen 0.57 length, 0.34 width. Habitus as in Fig. 1A, C, E. *Carapace*: pale orange, with brown, egg-shaped patches behind eyes, ovoid in dorsal view, strongly elevated in lateral view, surface of elevated portion of pars cephalica smooth, sides finely reticulate, fovea absent, lateral margin straight, smooth (Fig. 1B, D). *Clypeus*: straight in frontal view, vertical in lateral view, ALE separated from edge of carapace by their radius or more. *Eyes*: six, well developed, ALE largest, ALE circular, PME and PLE oval, posterior eye row procurved from both above and front, ALE separated by less than their radius, ALE-PLE separated by less than ALE radius, PME touching, PLE-PME touching (Fig. 1I). *Sternum*: longer than wide, pale orange, uniform, not fused to carapace, surface smooth, setae sparse. *Mouthparts*: chelicerae, endites and labium orange. Chelicerae straight, with finger-shaped sclerotized process (fsp) at base of fangs (Fig. 3H), proximal part of paturon with a thorn-like protrusion (tlp) (Figs 1G, H, 3G), fang groove with a few small and two larger denticles. Labium rectangular, fused to sternum, anterior margin not indented at middle. Anteromedian tip of endites with one strong, tooth-like projection (Fig. 1E, F). *Abdomen*: ovoid, rounded posteriorly. Posterior spiracles not connected by groove. Pedicel tube short, ribbed, scutum not extending far dorsal of pedicel. Dorsal scutum well sclerotized, pale orange, covering whole abdomen width and approximately 4/5 of abdomen length, fused to epigastric scutum, middle surface and sides smooth. Epigastric and postepigastric scutum well sclerotized, pale orange, fused, without posteriorly directed lateral apodemes. Dorsum setae present, light, needle-like. *Legs*: pale orange, femur I with two prolateral and two small retrolateral spines, tibia I with four pairs, metatarsus I with two pairs of long ventral spines. Leg II spination is similar to leg I except femur with only one prolateral and one retrolateral spine. Legs III and IV spineless. *Genitalia*: epigastric region with sperm pore middle sized, circular, situated at level of anterior spiracles. Palp strongly sclerotized, right and left palps symmetrical, trochanter with ventral projection (vp) (Fig. 3A, D), cymbium brown, fused with bulb, bulb brown, more than two times as long as cymbium, tapering apically, with two small ventral protuberances (vpr) (Fig. 3D), distal part elongated, with membranous leaf-shaped apophyses (lsa) (Fig. 3B, C, E, F).

Female (paratype). Total length 1.37; carapace 0.64 length, 0.51 width; abdomen 0.76 length, 0.53 width. Habitus as in Fig. 2A, C, E. As in male except as noted. *Carapace*: without any pattern. *Mouthparts*: chelicerae and endites unmodified. *Abdomen*: dorsal scutum covering less than 1/2 of abdomen length, less than 1/3 of abdomen width. Postepigastric scutum rectangular. *Genitalia*: the posterior margin of the epigastric scutum is lined with numerous needle-like setae. The epigastric groove is narrow. From the middle of the slightly thickened margin of the postepigastric scutum runs a dark, winding tube posteriorly (wt) (Fig. 2G, I), ending in an equilateral triangular shaped atrium (tsa) (Fig. 2H, J).

#### Distribution.

Singapore.

### Ischnothyreus brunneus

Taxon classificationAnimaliaAraneaeOonopidae

Tong & Li sp. n.

http://zoobank.org/14137BDE-A1CF-4315-8BF0-3C405C84C437

Figs 4 , 5 , 6

#### Type material.

**Holotype**: male (LKCNHM), Singapore: Central Catchment Nature Reserve (off Mandai Lake Road), Alt. 39 m, 1°24'30.7"N, 103°46'51.3"E, August 31, 2015, S. Li and Y. Tong leg. **Paratypes**: 7 males, 8 females (LKCNHM), same data as holotype.

#### Other material studied.

8 males, 14 females (SYNU-65), Singapore: Central Catchment Nature Reserve (off Mandai Lake Road), Alt. 39 m, 1°24'30.7"N, 103°46'51.3"E, August 31, 2015, S. Li and Y. Tong leg.; 3 females (SYNU-66), Singapore: Pulau Ubin, Alt. 2 m, 1°25'18.0"N, 103°56'25.4"E, August 22, 2015, S. Li and Y. Tong leg.;1 female (SYNU-67), Singapore: Central Catchment Nature Reserve (off Mandai Lake Road), Alt. 39 m, 1°24'30.7"N, 103°46'51.3"E, August 31, 2015, S. Li and Y. Tong leg.

#### Etymology.

The specific epithet means “brown” in Latin, and refers to the body color of this species; adjective.

#### Diagnosis.

Males of the new species is similar to those of *Ischnothyreus dactylinus* sp. n., but can be distinguished from it by the larger eyes and the unmodified chelicerae. Furthermore the distal part of the male palpal bulb lacks the finger-like apophyses present in *Ischnothyreus dactylinus* (Fig. 6A–F). Females of the new species is similar to those of *Ischnothyreus barus* Kranz-Baltensperger, 2011, but can be distinguished from it by the brown body color and the dark brown pattern on leg IV, and the small bell-shaped atrium (bsa) in the epigastric region (Fig. 5J).

**Figure 4.**
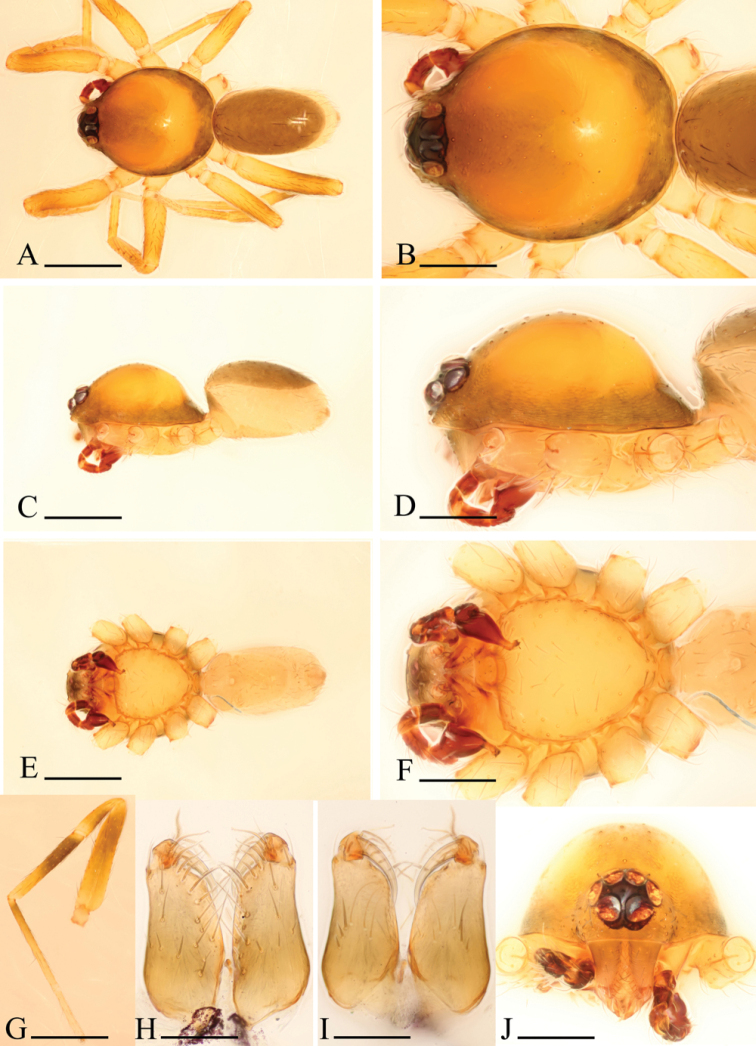
*Ischnothyreus brunneus* sp. n., male. **A, C, E** habitus, dorsal, lateral and ventral views **B, D, F, J** prosoma, dorsal, lateral, ventral and anterior views **G**, left leg IV, retrolateral view **H, I** chelicerae, anterior and posterior views. Scale bars: **A, C, E, G** = 0.4 mm; **B, D, F, J** = 0.2 mm; **H, I** = 0.1 mm.

**Figure 5.**
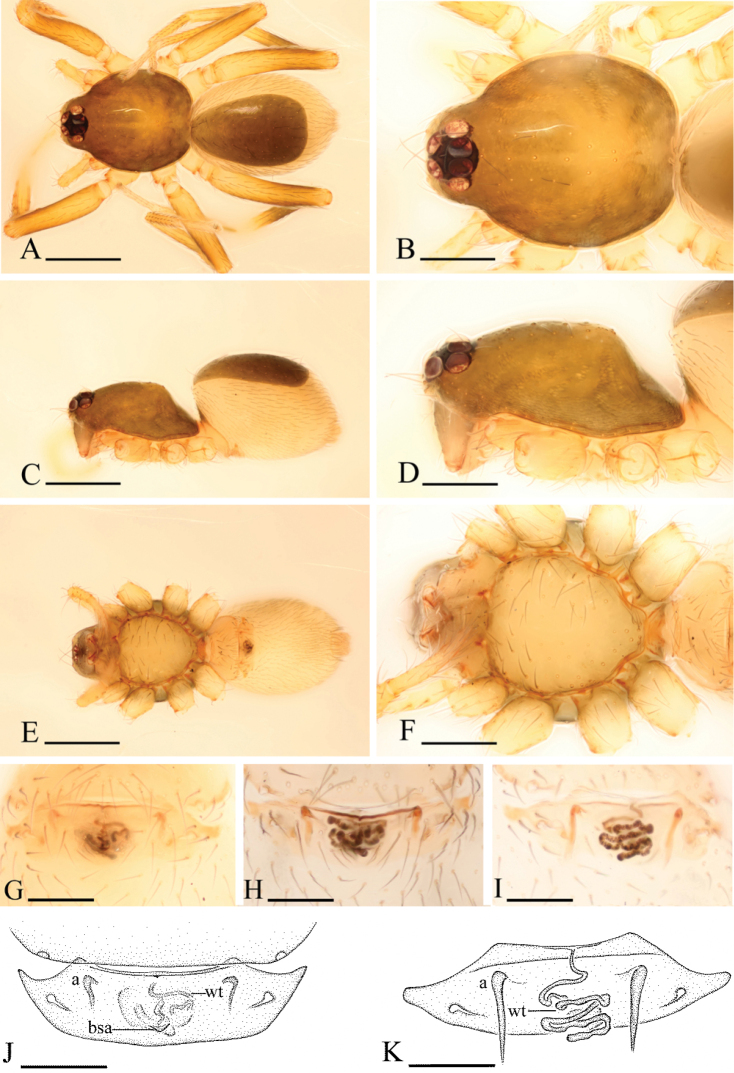
*Ischnothyreus brunneus* sp. n., female. **A, C, E** habitus, dorsal, lateral and ventral views **B, D, F** prosoma, dorsal, lateral and ventral views **G, H, J** epigastric region, ventral view **I, K** epigastric region, dorsal view (H, I cleared in lactic acid). Abbreviations: a = apodeme; bsa = bell-shaped atrium; wt = winding tube. Scale bars: **A, C, E** = 0.4 mm; **B, D, F** = 0.2 mm; **G–K** = 0.1 mm.

**Figure 6.**
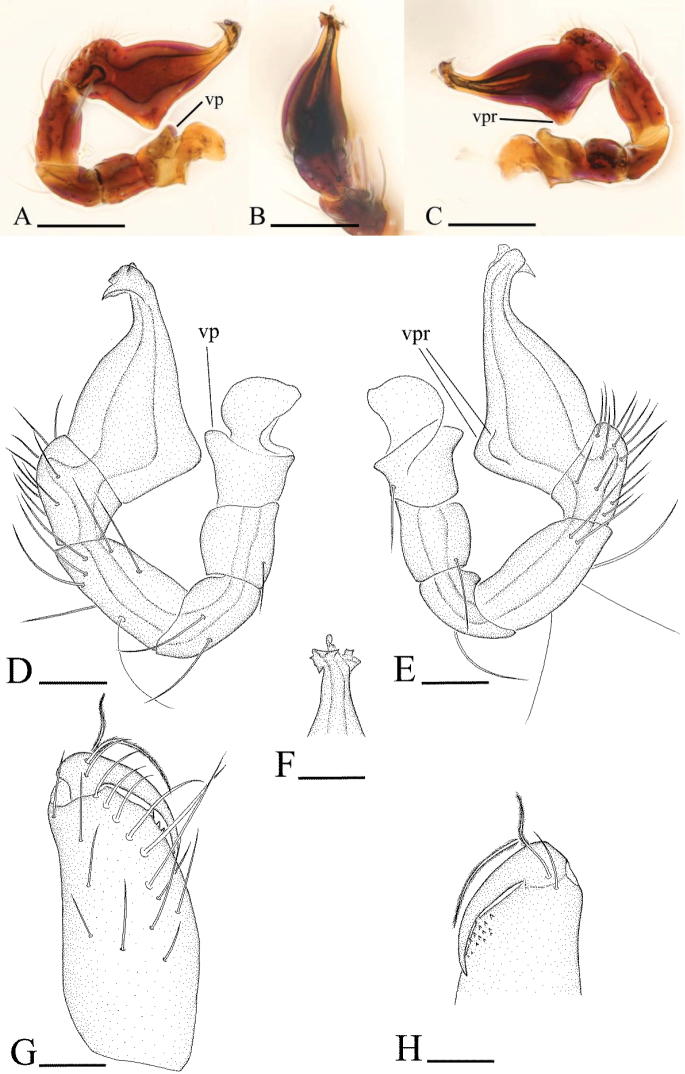
*Ischnothyreus brunneus* sp. n., male. **A, D** left palp, prolateral view **B** left palp, dorsal view **C, E** left palp, retrolateral view **F** distal part of palpal bulb, dorsal view **G, H** left chelicera, anterior and posterior views. Abbreviations: vp = ventral projection; vpr = ventral protuberance. Scale bars: **A–C** = 0.1 mm; **D–H** = 0.05 mm.

#### Description.

Male (holotype). Total length 1.36; carapace 0.73 length, 0.57 width; abdomen 0.67 length, 0.48 width. Habitus as in Fig. 4A, C, E. *Carapace*: yellow, dark brown on lateral and posterior surfaces, with brown egg-shaped patches behind eyes, ovoid in dorsal view, slightly elevated in lateral view, surface of elevated portion of pars cephalica smooth, sides finely reticulate, fovea absent, lateral margin straight, smooth (Fig. 4B, D). *Clypeus*: straight in frontal view, vertical in lateral view, high, ALE separated from edge of carapace by more than their radius. *Eyes*: six, well developed, ALE largest, ALE circular, PME and PLE oval, posterior eye row straight from above, procurved from front, ALE separated by less than their radius, ALE-PLE separated by less than ALE radius, PME touching, PLE-PME touching (Fig. 4J). *Sternum*: longer than wide, pale yellow, uniform, not fused to carapace, surface smooth, setae sparse. *Mouthparts*: chelicerae, endites, and labium yellow. Chelicerae straight, base of fangs unmodified, fang groove with many small denticles (Fig. 6G, H). Labium rectangular, fused to sternum, anterior margin not indented at middle. Anteromedian tip of endites with one strong, tooth-like projection (Fig. 4E, F). *Abdomen*: ovoid, rounded posteriorly. Posterior spiracles not connected by groove. Pedicel tube short, ribbed, scutum not extending far dorsal of pedicel. Dorsal scutum well sclerotized, dark brown, covering whole abdomen width and approximately 5/6 of abdomen length, fused to epigastric scutum, middle surface and sides smooth. Epigastric and postepigastric scutum well sclerotized, fused, upper pedicel tube region dark brown, the other part yellow, without posteriorly directed lateral apodemes. Dorsum setae present, light, needle-like. *Legs*: yellow, with dark brown pattern on distal part of femur IV and middle part of tibia IV (Fig. 4G), femur I with two prolateral and two small retrolateral spines, tibia I with four pairs, metatarsus I with two pairs of long ventral spines. Leg II spination is similar to leg I except femur with only one prolateral and one retrolateral spine. Legs III and IV spineless. *Genitalia*: epigastric region with sperm pore large, circular, situated at level of anterior spiracles. Palp strongly sclerotized, right and left palps symmetrical, trochanter with ventral projection (vp) (Fig. 6A, D), cymbium brown, fused with bulb, bulb brown, more than two times as long as cymbium, tapering apically, with two small ventral protuberances (vpr) (Fig. 6E), distal part elongated, with membranous outgrowth (Fig. 6B, C).

Female (paratype). Total length 1.47; carapace 0.76 length, 0.56 width; abdomen 0.79 length, 0.52 width. Habitus as in Fig. 5A, C, E. As in male except as noted. *Carapace*: dark brown, without any pattern. *Mouthparts*: endites unmodified. *Abdomen*: dorsal scutum covering approximately 2/3 of abdomen length, 1/2 of abdomen width. *Genitalia*: from the middle of the slightly thickened margin of the postepigastric scutum runs a dark, winding tube posteriorly (wt) (Fig. 5G, H), ending in a small bell-shaped atrium (bsa) (Fig. 5J).

#### Distribution.

Singapore.

### Ischnothyreus dactylinus

Taxon classificationAnimaliaAraneaeOonopidae

Tong & Li sp. n.

http://zoobank.org/FA51E4D1-06C2-413E-A767-8B380A779DAF

Figs 7 , 8 , 9

#### Type material.

**Holotype**: male (LKCNHM), Singapore: Central Catchment Nature Reserve, near Singapore Zoo, Alt. 50 m, 1°24'22.3"N, 103°47'7.4"E, August 30, 2015, S. Li and Y. Tong leg. **Paratypes**: 5 males, 8 females (LKCNHM), same data as holotype.

#### Other material studied.

1 male, 1 female (SYNU-85), Singapore: Bukit Timah Nature Reserve, Alt. 86 m, 1°21'37.4"N, 103°46'30.0"E, August 24, 2015, S. Li and Y. Tong leg.; 7 males, 9 females (SYNU-86), Singapore: Central Catchment Nature Reserve, near Singapore Zoo, Alt. 50 m, 1°24'22.3"N, 103°47'7.4"E, August 30, 2015, S. Li and Y. Tong leg.; 1 male, 8 females (SYNU-87), Singapore: Central Catchment Nature Reserve (off Mandai Lake Road), Alt. 39 m, 1°24'30.7"N, 103°46'51.3"E, August 31, 2015, S. Li and Y. Tong leg.; 1 female (SYNU-88), Singapore: Central Catchment Nature Reserve, near Mandai Agrotechnology Park, Alt. 46 m, 1°24'53.7"N, 103°47'56.2"E, Sep 1, 2015, S. Li and Y. Tong leg.; 1 male, 1 female (SYNU-89), Singapore: Central Catchment Nature Reserve, Alt. 46 m, 1°21'13.3"N, 103°48'29.4"E, August 27, 2015, S. Li and Y. Tong leg.; 1 male, 1 female (SYNU-91), Singapore: Central Catchment Nature Reserve, Alt. 39 m, 1°21'17.9"N, 103°47'50.7"E, August 25, 2015, S. Li and Y. Tong leg.; 1 male (SYNU-92), Singapore: Central Catchment Nature Reserve, Alt. 60 m, 1°21'21.7"N, 103°48'3.8"E, August 26, 2015, S. Li and Y. Tong leg.; 2 females (SYNU-93), Singapore: Bukit Timah Nature Reserve, Bukit Timah Summit, Alt. 163 m, 1°21'16.65"N, 103°46'34.95"E, August 19, 2015, S. Li and Y. Tong leg.; 1 male, 1 female (SYNU-94), Singapore: Bukit Timah Nature Reserve, Bukit Timah Summit, Alt. 163 m, 1°21'16.65"N, 103°46'34.95"E, August 19, 2015, S. Li and Y. Tong leg.; 4 females (SYNU-95), Singapore: Bukit Timah Nature Reserve, Catchment Path, Alt. 107 m, 1°21'12.5"N, 103°46'50.6"E, August 20, 2015, S. Li and Y. Tong leg.; 1 male, 1 female (SYNU-96), Singapore: Bukit Timah Nature Reserve, Bukit Timah Summit, Alt. 163 m, 1°21'16.65"N, 103°46'34.95"E, August 19, 2015, S. Li and Y. Tong leg.; 2 males, 5 females (SYNU-97), Singapore: Central Catchment Nature Reserve, near Singapore Zoo, Alt. 50 m, 1°24'22.3"N, 103°47'7.4"E, August 30, 2015, S. Li and Y. Tong leg.; 1 male, 3 females (SYNU-98), Singapore: Bukit Timah Nature Reserve, Seraya Loop, Alt. 118 m, 1°21'25.4"N, 103°46'25.3"E, August 17, 2015, S. Li and Y. Tong leg.

#### Etymology.

The specific epithet means “finger-like” in Greek, and refers to the long apophysis on the distal part of the male papal bulb (Fig. 9D, E); adjective.

#### Diagnosis.

The new species is similar to *Ischnothyreus browni* Chickering, 1968 (Platnick et al. 2012), but can be distinguished from it by the dark brown body color, the flake-like dorsal process (fdp) on the male chelicerae (Fig. 9F, G), the finger-like apophysis on the distal part of the male papal bulb (Fig. 9D, E) and the fan-shaped atrium (fsa) in the female epigastric region (Fig. 8H). The female epigastric region of the new species is also similar to that of *Ischnothyreus balu* Kranz-Baltensperger, 2011, but can be distinguished from it by the larger abdominal scutum and the color patterns on legs and abdomen.

#### Description.

**Figure 7.**
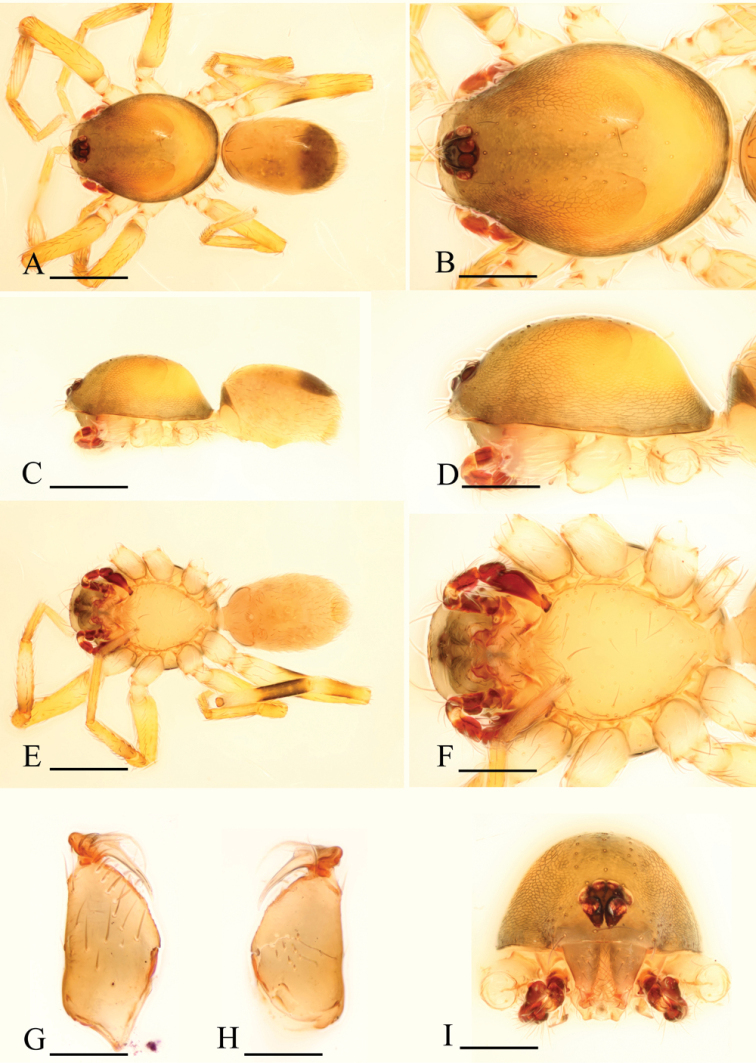
*Ischnothyreus dactylinus* sp. n., male. **A, C, E** habitus, dorsal, lateral and ventral views **B, D, F, I** prosoma, dorsal, lateral, ventral and anterior views **G, H** left chelicera, anterior and posterior views. Scale bars: **A, C, E** = 0.4 mm; **B, D, F, I** = 0.2 mm; **G, H** = 0.1 mm.

Male (holotype). Total length 1.43; carapace 0.78 length, 0.57 width; abdomen 0.65 length, 0.39 width. Habitus as in Fig. 7A, C, E. *Carapace*: yellow, dark brown on lateral and posterior surfaces, with brown egg-shaped patches behind eyes, ovoid in dorsal view, slightly elevated in lateral view, surface of elevated portion of pars cephalica smooth, sides strongly reticulate, fovea absent, lateral margin straight, smooth (Fig. 7B, D). *Clypeus*: straight in frontal view, vertical in lateral view, high, ALE separated from edge of carapace by more than twice of their diameter. *Eyes*: six, very small, ALE largest, ALE circular, PME and PLE oval, posterior eye row procurved from both above and front, ALE separated by less than their radius, ALE-PLE separated by less than ALE radius, PME touching, PLE-PME touching (Fig. 7I). *Sternum*: longer than wide, pale yellow, uniform, not fused to carapace, surface smooth, setae sparse. *Mouthparts*: chelicerae, endites and labium yellow. Chelicerae straight, base of fangs with a flake-like dorsal process (fdp) (Fig. 9F, G), fang groove with a few small and one larger denticles. Labium rectangular, fused to sternum, anterior margin not indented at middle. Anteromedian tip of endites with one strong, tooth-like projection (Fig. 7E, F). *Abdomen*: ovoid, rounded posteriorly. Posterior spiracles not connected by groove. Pedicel tube short, ribbed, scutum not extending far dorsal of pedicel. Dorsal scutum well sclerotized, yellow, except dark brown on posterior part, covering, whole abdomen width and approximately 5/6 of abdomen length, not fused to epigastric scutum, middle surface and sides smooth. Epigastric and postepigastric scutum well sclerotized, fused, the upper part of the pedicel tube is dark brown, the other part pale yellow, without posteriorly directed lateral apodemes. Dorsum setae present, light, needle-like. *Legs*: yellow, with dark brown pattern on subbasal part of femur, trochanter and basal half part of tibia of leg IV, femur I with two prolateral and two small retrolateral spines, tibia I with four pairs, metatarsus I with two pairs of long ventral spines. Leg II spination is similar to leg I except femur with only one prolateral and one retrolateral spine. Legs III and IV spineless. *Genitalia*: epigastric region with sperm pore large, circular, situated at level of anterior spiracles. Palp strongly sclerotized, right and left palps symmetrical, proximal segments brown, trochanter with ventral projection (vp) (Fig. 9A), cymbium brown, fused with bulb, bulb brown, more than two times as long as cymbium, tapering apically, with one large ventral protuberance (vpr) (Fig. 9B, C), distal part elongated, with two long apophyses, one sclerotized, finger-like (sfa), one membranous, triangle-shaped (mta) (Fig. 9D, E).

**Figure 8.**
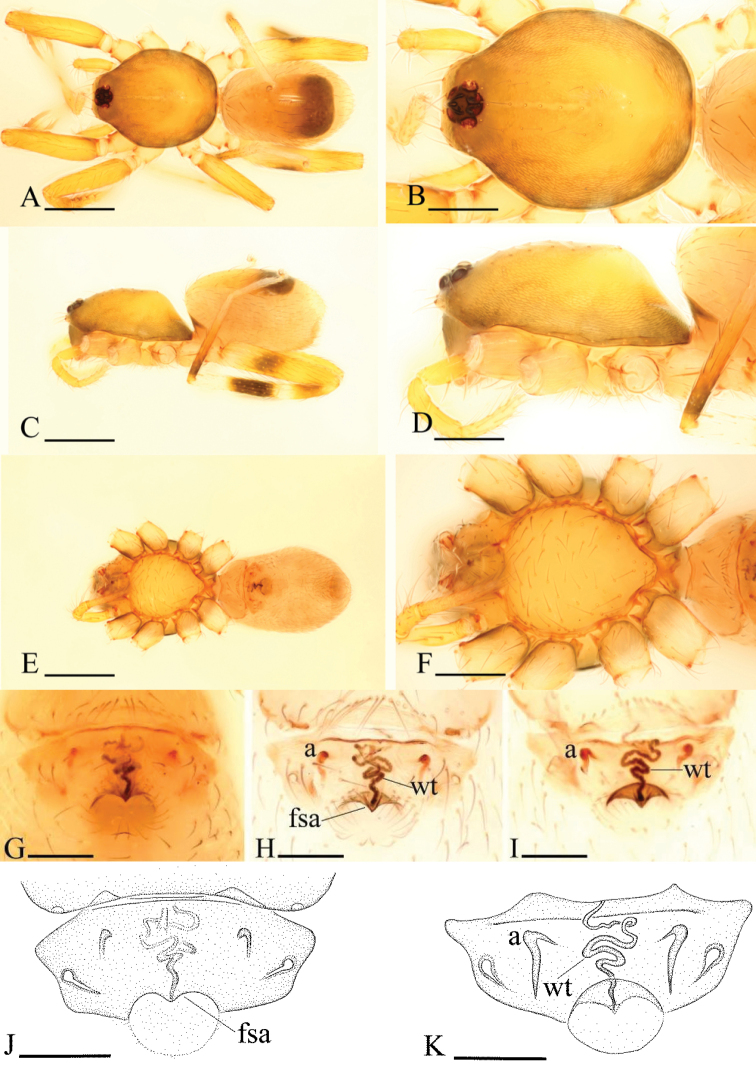
*Ischnothyreus dactylinus* sp. n., female. **A, C, E** habitus, dorsal, lateral and ventral views **B, D, F** prosoma, dorsal, lateral and ventral views **G, H, J** epigastric region, ventral view **I, K** epigastric region, dorsal view (H, I cleared in lactic acid). Abbreviations: a = apodeme; fsa = fan-shaped atrium; wt = winding tube. Scale bars: **A, C, E** = 0.4 mm; **B, D, F** = 0.2 mm; **G–K** = 0.1 mm.

**Figure 9.**
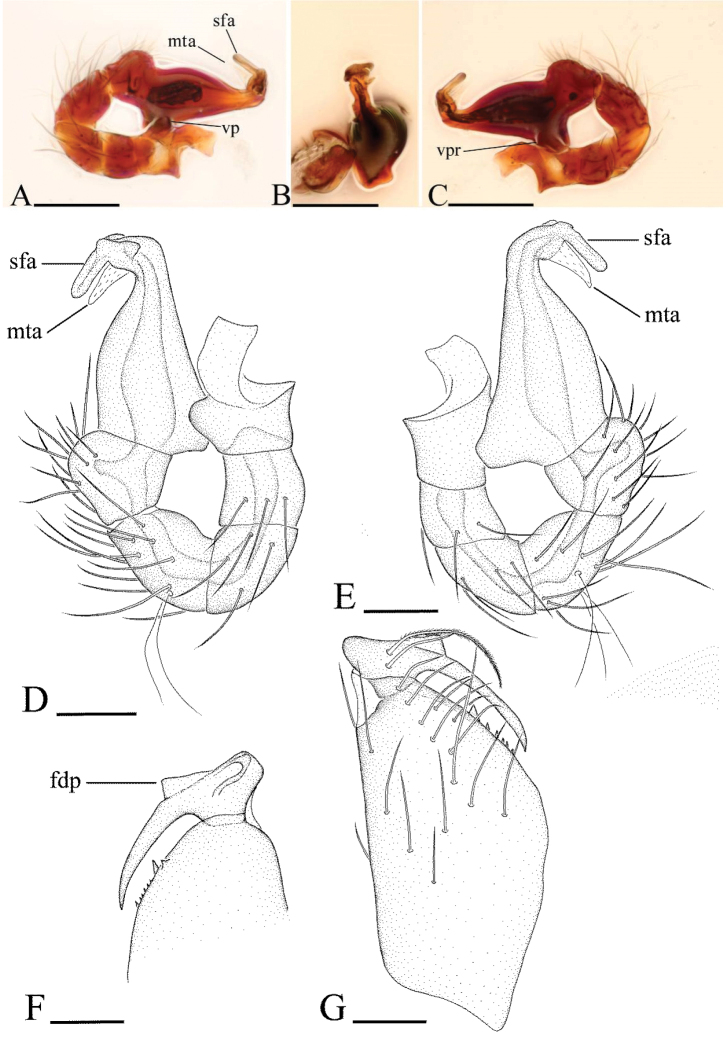
*Ischnothyreus dactylinus* sp. n., male. **A, D** left palp, prolateral view **B** distal part of palpal bulb, apical view **C, E** left palp, retrolateral view **F**, **G** left chelicera, posterior and anterior views. Abbreviations: fdp = flake-like dorsal process; mta = membranous, triangle-shaped apophysis; sfa = sclerotized, finger-like apophysis; vp = ventral projection; vpr = ventral protuberance. Scale bars: **A–C** = 0.1 mm; **D–G** = 0.05 mm.

Female (paratype). Total length 1.51; carapace 0.73 length, 0.58 width; abdomen 0.76 length, 0.49 width. Habitus as in Fig. 8A, C, E. As in male except as noted. *Carapace*: without any pattern. *Mouthparts*: endites unmodified. *Abdomen*: dorsal scutum covering less than 5/6 of abdomen length, 2/3 of abdomen width. Postepigastric scutum widely hexagonal. *Genitalia*: the posterior margin of the epigastric scutum is lined with numerous needle-like setae. The epigastric groove is narrow. From the middle of the slightly thickened margin of the postepigastric scutum runs a dark, winding tube posteriorly (wt) (Fig. 8G, J), ending in a fan-shaped atrium (fsa) (Fig. 8H).

#### Distribution.

Singapore.

### Ischnothyreus poculum

Taxon classificationAnimaliaAraneaeOonopidae

Tong & Li sp. n.

http://zoobank.org/352FE6DD-AD01-432A-8ED0-5822A77DB4D6

Figs 10 , 11 , 12

#### Type material.

**Holotype**: male (LKCNHM), Singapore: Central Catchment Nature Reserve, near Singapore Zoo, Alt. 50 m, 1°24'22.3"N, 103°47'7.4"E, August 30, 2015, S. Li and Y. Tong leg. **Paratypes**: 1 female (LKCNHM), same data as holotype; 1 female (LKCNHM), Singapore: Bukit Timah Nature Reserve, Alt. 86 m, 1°21'37.4"N, 103°46'30.0"E, August 24, 2015, S. Li and Y. Tong leg.

#### Other material studied.

2 females (SYNU-71), Singapore: Central Catchment Nature Reserve, near Mandai Agrotechnology Park, Alt. 46 m, 1°24'53.7"N, 103°47'56.2"E, Sep 1, 2015, S. Li and Y. Tong leg.; 1 female (SYNU-72), Singapore: Central Catchment Nature Reserve, Alt. 46 m, 1°21'13.3"N, 103°48'29.4"E, August 27, 2015, S. Li and Y. Tong leg.

#### Etymology.

The specific epithet means “bowl” in Latin, and refers to the bowl-shaped atrium in the female epigastric region; noun.

#### Diagnosis.

**Figure 10.**
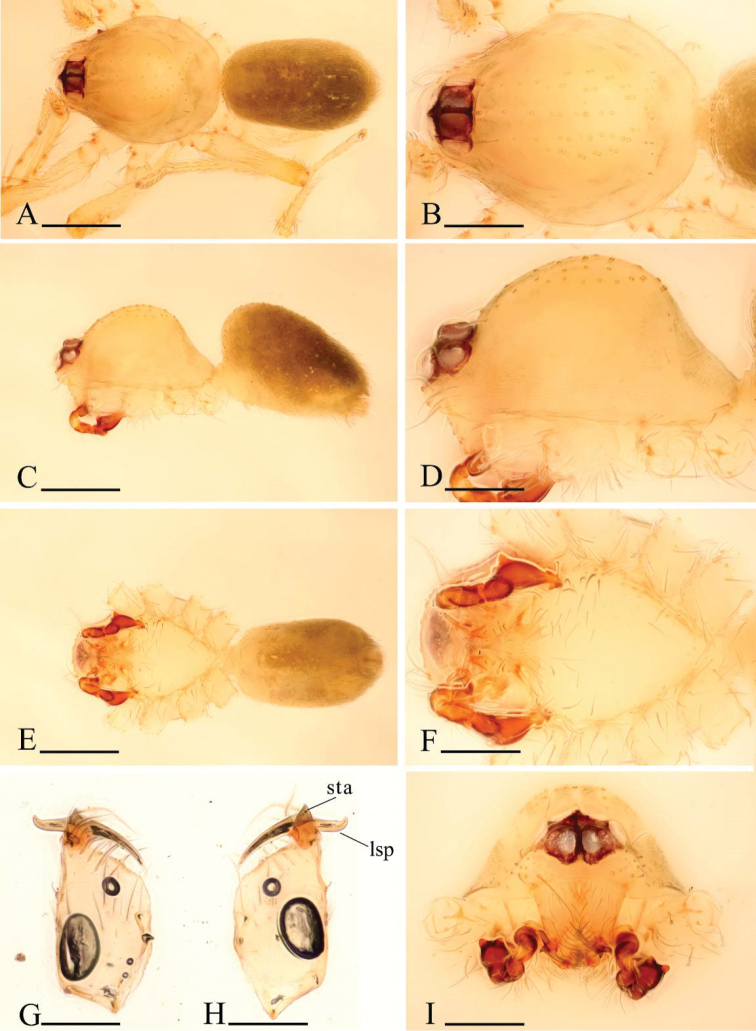
*Ischnothyreus poculum* sp. n., male. **A, C, E** habitus, dorsal, lateral and ventral views **B, D, F, I** prosoma, dorsal, lateral, ventral and anterior views **G, H** left chelicerae, anterior and posterior views. Abbreviations: lsp = long sclerotized process; sta = sclerotized triangular-shaped apophysis. Scale bars: **A, C, E** = 0.4 mm; **B, D, F, I** = 0.2 mm; **G, H** = 0.1 mm.

**Figure 11.**
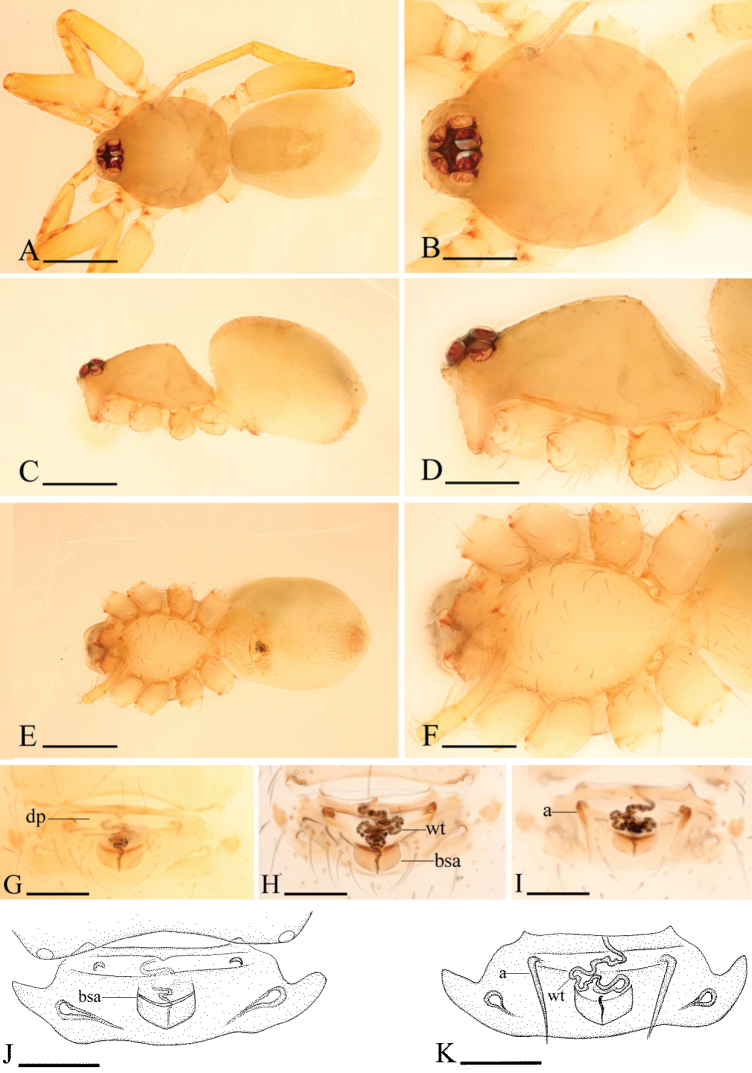
*Ischnothyreus poculum* sp. n., female. **A, C, E** habitus, dorsal, lateral and ventral views **B, D, F** prosoma, dorsal, lateral and ventral views **G, H, J** epigastric region, ventral view **I, K** epigastric region, dorsal view (H, I cleared in lactic acid). Abbreviations: a = apodeme; bsa = bowl-shaped atrium; dp = depression; wt = winding tube. Scale bars: **A, C, E** = 0.4 mm; **B, D, F** = 0.2 mm; **G–K** = 0.1 mm.

**Figure 12.**
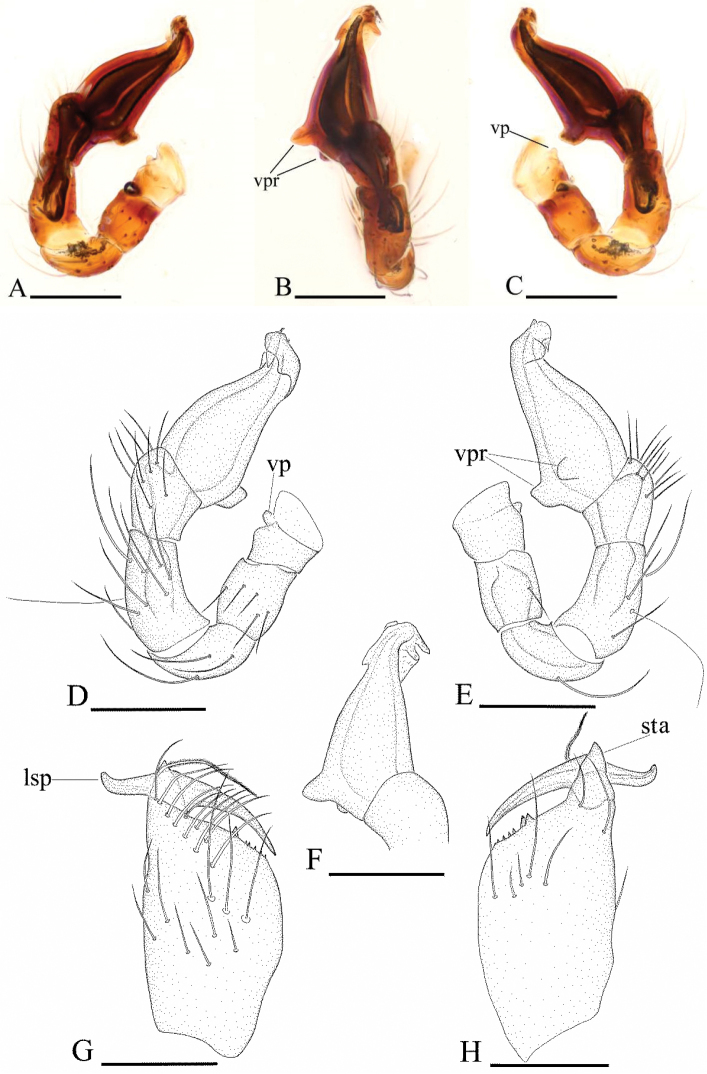
*Ischnothyreus poculum* sp. n., male. **A, D** left palp, prolateral view **B, F** left palpal bulb, dorsal view **C**, **E** left palp, retrolateral view **G, H** left chelicerae, anterior and posterior views. Abbreviations: lsp = long sclerotized process; sta = sclerotized triangular-shaped apophysis; vp = ventral projection; vpr = ventral protuberance. Scale bars: 0.1 mm.

The new species is similar to *Ischnothyreus campanaceus* Tong & Li, 2008, but can be distinguished from it by the small abdominal dorsal scutum, the long sclerotized process (lsp) and small sclerotized triangular-shaped apophysis (sta) at base of fangs in male (Fig. 10H), and the bowl-shaped atrium in the female epigastric region (Fig. 11G–K). Males of the new species is also similar to those of *Ischnothyreus jojo* Kranz-Baltensperger, 2011 by the long sclerotized process (lsp) on the cheliceral fang (Figs 10G, H, 12G), but can be distinguished from it by the small sclerotized triangular-shaped apophysis (sta) at base of fangs (Fig. 10H) and the membranous outgrowth on distal part of male palp (Fig. 12A–E).

#### Description.

Male (holotype). Total length 1.64; carapace 0.83 length, 0.67 width; abdomen 0.80 length, 0.46 width. Habitus as in Fig. 10A, C, E. *Carapace*: pale orange, with brown egg-shaped patches behind eyes, ovoid in dorsal view, strongly elevated in lateral view, surface of elevated portion of pars cephalica smooth, sides finely reticulate, fovea absent, lateral margin straight, smooth (Fig. 10B, D). *Clypeus*: straight in frontal view, vertical in lateral view, ALE separated from edge of carapace by their radius or more. *Eyes*: six, well developed, ALE largest, ALE circular, PME and PLE oval, posterior eye row straight from above, procurved from front, ALE separated by less than their radius, ALE-PLE separated by less than ALE radius, PME touching, PLE-PME touching (Fig. 10I). *Sternum*: longer than wide, pale orange, uniform, not fused to carapace, surface smooth, setae sparse. *Mouthparts*: chelicerae, endites and labium orange. Chelicerae straight, with long sclerotized process (lsp) and small sclerotized triangular-shaped apophysis (sta) at base of fangs (Fig. 12G, H), fang groove with a few small and one larger denticles. Labium rectangular, fused to sternum, anterior margin not indented at middle. Anteromedian tip of endites with one strong, tooth-like projection (Fig. 10E, F). *Abdomen*: ovoid, rounded posteriorly. Posterior spiracles not connected by groove. Pedicel tube short, ribbed, scutum not extending far dorsal of pedicel. Dorsal scutum weakly sclerotized, pale orange, covering approximately 1/2 of abdomen length, 1/2 of abdomen width, fused to epigastric scutum. Epigastric and postepigastric scutum weakly sclerotized, pale orange, fused, without posteriorly directed lateral apodemes. Dorsum setae present, light, needle-like. *Legs*: pale orange, femur I with two prolateral and two small retrolateral spines, tibia I with four pairs, metatarsus I with two pairs of long ventral spines. Leg II spination is similar to leg I except femur with only one prolateral and one retrolateral spine. Legs III and IV spineless. *Genitalia*: epigastric region with sperm pore middle sized, circular, situated at level of anterior spiracles. Palp strongly sclerotized, right and left palps symmetrical, trochanter with ventral projection (vp) (Fig. 12C, D), cymbium brown, fused with bulb, bulb brown, more than two times as long as cymbium, tapering apically, with two ventral protuberances (vpr) (Fig. 12B), distal part elongated, with membranous outgrowth (Fig. 12D, E, F).

Female (paratype). Total length 1.55; carapace 0.73 length, 0.61 width; abdomen 0.82 length, 0.59 width. Habitus as in Fig. 11A, C, E. As in male except as noted. *Carapace*: without any pattern. *Mouthparts*: chelicerae and endites unmodified. *Abdomen*: dorsal scutum well sclerotized, postepigastric scutum boat-shaped, very narrow. *Genitalia*: anterior margin of the postepigastric scutum slightly sclerotized, behind the anterior margin is a depression (dp); the winding tube runs posteriorly (wt), ending in a bowl-shaped atrium (bsa) (Fig. 11G–K).

#### Distribution.

Singapore.

### Ischnothyreus tectorius

Taxon classificationAnimaliaAraneaeOonopidae

Tong & Li sp. n.

http://zoobank.org/8044453E-C913-4CB4-A9C0-4179733324F6

Figs 13 , 14 , 15

#### Type material.

**Holotype**: male (LKCNHM), Singapore: Central Catchment Nature Reserve, near Mandai Agrotechnology Park, Alt. 46 m, 1°24'53.7"N, 103°47'56.2"E, Sep 1, 2015, S. Li and Y. Tong leg. **Paratypes**: 7 males, 7 females (LKCNHM), same data as holotype.

#### Other material studied.

2 males, 1 female (SYNU-73), Singapore: Bukit Timah Nature Reserve, Alt. 86 m, 1°21'37.4"N, 103°46'30.0"E, August 24, 2015, S. Li and Y. Tong leg.; 7 males, 10 females (SYNU-74), Singapore: Central Catchment Nature Reserve, near Singapore Zoo, Alt. 50 m, 1°24'22.3"N, 103°47'7.4"E, August 30, 2015, S. Li and Y. Tong leg.; 12 females (SYNU-75), Singapore: Bukit Timah Nature Reserve, Seraya Loop, Alt. 118 m, 1°21'25.4"N, 103°46'25.3"E, August 17, 2015, S. Li and Y. Tong leg.; 6 males, 9 females (SYNU-76), Singapore: Central Catchment Nature Reserve (off Mandai Lake Road), Alt. 39 m, 1°24'30.7"N, 103°46'51.3"E, August 31, 2015, S. Li and Y. Tong leg.; 2 males, 5 females (SYNU-77), Singapore: Bukit Timah Nature Reserve, Catchment Path, Alt. 107 m, 1°21'12.5"N, 103°46'50.6"E, August 20, 2015, S. Li and Y. Tong leg.; 6 males, 11 females (SYNU-78), Singapore: Central Catchment Nature Reserve, near Singapore Zoo, Alt. 50 m, 1°24'22.3"N, 103°47'7.4"E, August 30, 2015, S. Li and Y. Tong leg.; 4 males, 3 females (SYNU-79), Singapore: Bukit Timah Nature Reserve, Jungle Fall Stream, Alt. 118 m, 1°21'25.4"N, 103°46'25.3"E, August 18, 2015, S. Li and Y. Tong leg.; 3 males, 3 females (SYNU-80), Singapore: Bukit Timah Nature Reserve, Jungle Fall Stream, Alt. 118 m, 1°21'25.4"N, 103°46'25.3"E, August 18, 2015, S. Li and Y. Tong leg.; 2 males, 2 females (SYNU-81), Singapore: Bukit Timah Nature Reserve, Jungle Fall Stream, Alt. 118 m, 1°21'25.4"N, 103°46'25.3"E, August 18, 2015, S. Li and Y. Tong leg.; 2 males, 2 females (SYNU-82), Singapore: Bukit Timah Nature Reserve, Bukit Timah Summit, Alt. 163 m, 1°21'16.65"N, 103°46'34.95"E, August 19, 2015, S. Li and Y. Tong leg.; 1 female (SYNU-83), Singapore: Bukit Timah Nature Reserve, Catchment Path, Alt. 107 m, 1°21'12.5"N, 103°46'50.6"E, August 20, 2015, S. Li and Y. Tong leg.; 2 females (SYNU-84), Singapore: Central Catchment Nature Reserve (off Mandai Lake Road), Alt. 39 m, 1°24'30.7"N, 103°46'51.3"E, August 31, 2015, S. Li and Y. Tong leg.; 2 males, 1 female (SYNU-90), Singapore: Pulau Ubin, Alt. 2 m, 1°25'18.0"N, 103°56'25.4"E, August 22, 2015, S. Li and Y. Tong leg.

#### Etymology.

The specific epithet means “usable to cover a roof” in Latin, and refers to the long protruding extension on male clypeus; adjective.

#### Diagnosis.

**Figure 13.**
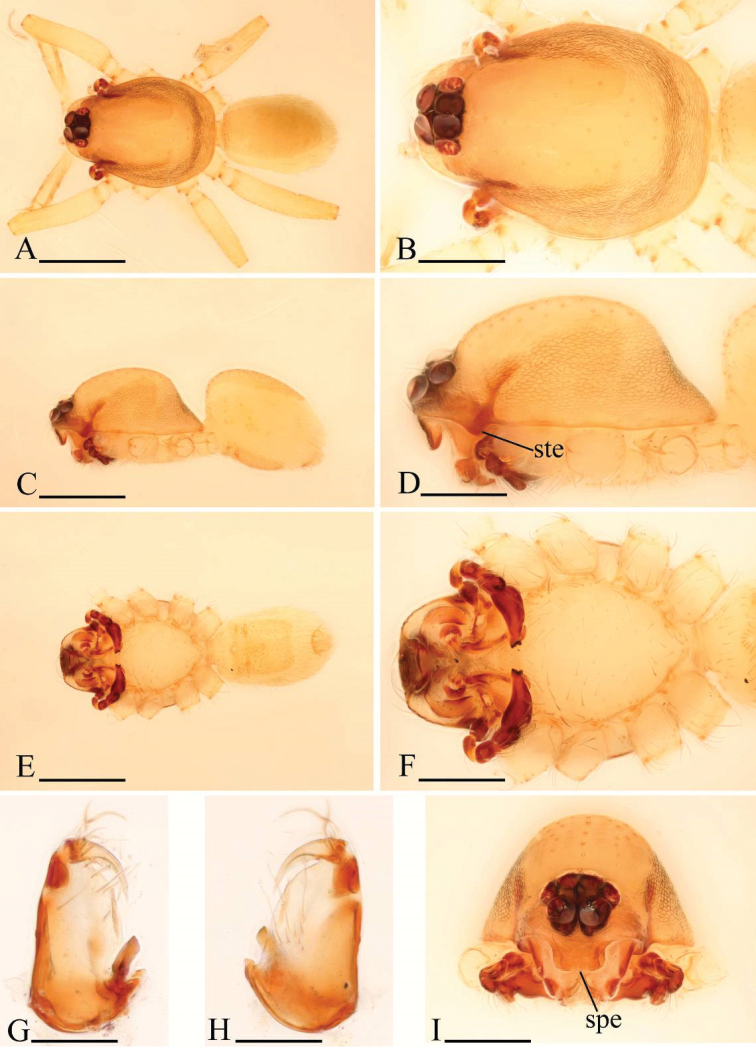
*Ischnothyreus tectorius* sp. n., male. **A, C, E** habitus, dorsal, lateral and ventral views **B, D, F, I** prosoma, dorsal, lateral, ventral and anterior views **G, H** left chelicera, anterior and posterior views. Abbreviations: spe = strongly protruding extension; ste = sclerotized, triangular extension. Scale bars: **A, C, E** = 0.4 mm; **B, D, F, I** = 0.2 mm; **G, H** = 0.1 mm.

**Figure 14.**
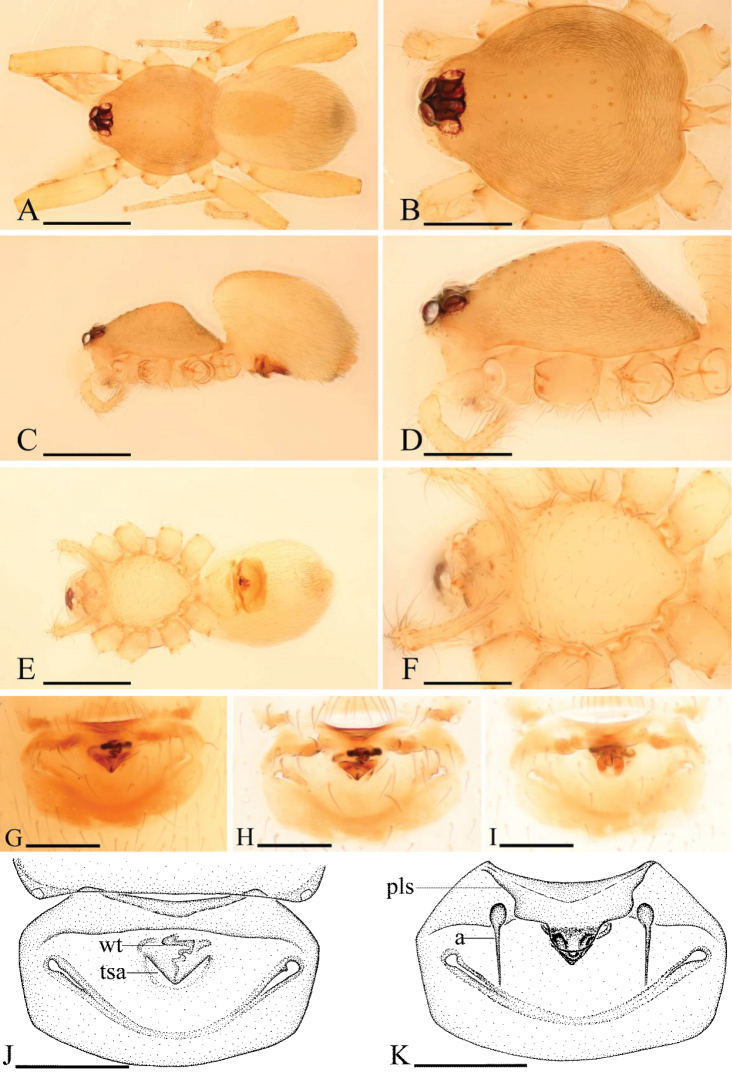
*Ischnothyreus tectorius* sp. n., female. **A, C, E** habitus, dorsal, lateral and ventral views **B, D, F** prosoma, dorsal, lateral and ventral views **G, H, J** epigastric region, ventral view **I, K** epigastric region, dorsal view (**H, I** cleared in lactic acid). Abbreviations: a = apodeme; pls = plate-like sclerite; tsa = triangular-shaped atrium; wt = winding tube. Scale bars: **A, C, E** =0.4 mm; **B, D, F** = 0.2 mm; **G–K** = 0.1 mm.

**Figure 15.**
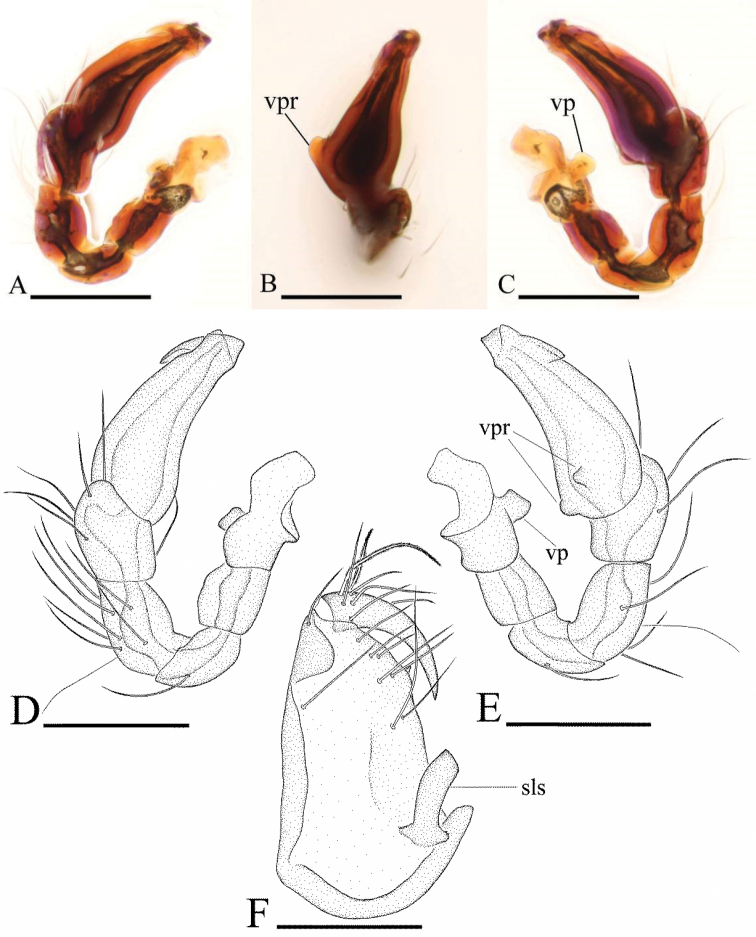
*Ischnothyreus tectorius* sp. n., male. **A, D** left palp, prolateral view **B** left palpal bulb, dorsal view **C, E** left palp, retrolateral view **F** left chelicerae, anterior view. Abbreviations: sls = scape-like sclerite; vp = ventral projection; vpr = ventral protuberance. Scale bars: 0.1 mm.

The new species can be distinguished from the congeneric species by the strongly protruding extension (spe) on male clypeus (Fig. 13I), the modifications on male chelicerae (Figs 13G, H, 15F) and the large, plate like sclerite (pls) in the female epigastric region (Fig. 14J, K).

#### Description.

Male (holotype). Total length 1.26; carapace 0.73 length, 0.57 width; abdomen 0.65 length, 0.35 width. Habitus as in Fig. 13A, C, E. *Carapace*: pale orange, with brown egg-shaped patches behind eyes, ovoid in dorsal view, strongly elevated in lateral view, surface of elevated portion of pars cephalica smooth, sides strongly reticulate, fovea absent, lateral margin straight, smooth (Fig. 13B, D). *Clypeus*: anterior margin with strongly protruding extension (spe) (Fig. 13I). Carapace anterolateral corners with strongly sclerotized, triangular extension (ste) (Fig. 13D). *Eyes*: six, well developed, ALE largest, ALE circular, PME and PLE oval, posterior eye row procurved from both above and front, ALE separated by less than their radius, ALE-PLE separated by less than ALE radius, PME touching, PLE-PME touching. *Sternum*: longer than wide, pale orange, uniform, not fused to carapace, surface smooth, setae sparse. *Mouthparts*: chelicerae, endites and labium orange. Chelicerae straight, base of fang unmodified, strongly sclerotized at lateral margin of paturon, proximal part of paturon with a scape-like sclerite (sls) (Fig. 15F), fang groove with a small denticle. Labium rectangular, fused to sternum, anterior margin not indented at middle. Anteromedian tip of endites with one strong, tooth-like projection (Fig. 13E, F). *Abdomen*: ovoid, rounded posteriorly. Posterior spiracles connected by groove. Pedicel tube short, ribbed, scutum not extending far dorsal of pedicel. Dorsal scutum well sclerotized, pale orange, covering approximately 4/5 of abdomen length, 2/3 of abdomen width, fused to epigastric scutum, middle surface and sides smooth. Epigastric and postepigastric scutum well sclerotized, pale orange, fused, without posteriorly directed lateral apodemes. Dorsum setae present, light, needle-like. *Legs*: pale orange, femur I with two prolateral and two small retrolateral spines, tibia I with four pairs, metatarsus I with two pairs of long ventral spines. Leg II spination is similar to leg I except femur with only one prolateral and one retrolateral spine. Legs III and IV spineless. *Genitalia*: epigastric region with sperm pore large, circular, situated at level of anterior spiracles, anterior margin of sperm pore with a fringe of needle-like setae. Palp strongly sclerotized, right and left palps symmetrical, proximal segments brown, trochanter with ventral projection (vp) (Fig. 15C), cymbium brown, fused with bulb, bulb brown, more than two times as long as cymbium, tapering apically, with two small ventral protuberance (vpr) (Fig. 15E), distal part elongated, end stout (Fig. 15A, C, D, E).

Female (paratype). Total length 1.24; carapace 0.72 length, 0.44 width; abdomen 0.77 length, 0.56 width. Habitus as in Fig. 14A, C, E. As in male except as noted. *Carapace*: without any pattern, broadly oval in dorsal view. *Clypeus*: margin unmodified, ALE separated from edge of carapace by less than their radius. *Mouthparts*: chelicerae and endites unmodified. *Abdomen*: dorsal scutum covering less than 1/2 of abdomen length, less than 1/3 of abdomen width. Postepigastric scutum rectangular, strongly sclerotized. *Genitalia*: the posterior margin of the epigastric scutum is lined with numerous needle-like setae. The epigastric groove is narrow. From the middle of the strongly thickened margin of the postepigastric scutum runs a dark, winding tube posteriorly (wt), ending in a triangular-shaped atrium (tsa) (Fig. 14J); from dorsal view, a large, plate like sclerite (pls) covers the internal structures (Fig. 14K).

#### Distribution.

Singapore.

## Supplementary Material

XML Treatment for Ischnothyreus an

XML Treatment for Ischnothyreus brunneus

XML Treatment for Ischnothyreus dactylinus

XML Treatment for Ischnothyreus poculum

XML Treatment for Ischnothyreus tectorius
